# Equipping Learners to Evaluate Online Health Care Resources: Longitudinal Study of Learning Design Strategies in a Health Care Massive Open Online Course

**DOI:** 10.2196/15177

**Published:** 2020-02-26

**Authors:** Louise M Blakemore, Sarah E M Meek, Leah K Marks

**Affiliations:** 1 National Heart and Lung Institute Imperial College London London United Kingdom; 2 School of Medicine, Dentistry and Nursing University of Glasgow Glasgow United Kingdom

**Keywords:** health care education, learning analytics, MOOC, plagiarism, ehealth, eHealth literacy, digital health literacy, misinformation, assessment, digital literacy

## Abstract

**Background:**

The digital revolution has led to a boom in the number of available online health care resources. To navigate these resources successfully, digital literacy education is required. Learners who can evaluate the reliability and validity of online health care information are likely to be more effective at avoiding potentially dangerous misinformation. In addition to providing health care education, massive open online courses (MOOCs) are well positioned to play a role in providing digital literacy education in this context.

**Objective:**

This study focused on learners enrolled in a MOOC on cancer genomics. The aim of this study was to evaluate the efficacy of a series of digital literacy–related activities within this course. This was an iterative study, with changes made to digital literacy–related activities in 4 of the 8 runs of the course.

**Methods:**

This mixed methods study focused on learner engagement with the digital literacy–related activities, including the final course written assignment. Quantitative data including the number of references listed in each written assignment were compared between successive runs. Qualitative data in the form of learner comments on discussion forums for digital literacy–related tasks were evaluated to determine the impact of these educational activities.

**Results:**

Using the number of references included for each final course assignment as an indicator of digital literacy skills, the digital literacy–related activities in the final 2 runs were judged to be the most successful. We found a statistically significant increase in the number of references cited by learners in their final written assignments. The average number of references cited in Run 8 was significantly higher (3.5) than in Run 1 (1.8) of the MOOC (*P*=.001). Learner comments in Runs 7 and 8 showed that a poll in which learners were asked to select which of 4 online resources was reliable was effective in stimulating learner discussion about how to evaluate resource reliability.

**Conclusions:**

Similar to many health care MOOCs, the course studied here had a heterogeneous group of learners, including patients (and their families), the public, health care students, and practitioners. Carefully designing a range of digital literacy–related activities that would be beneficial to this heterogenous group of learners enabled learners to become more effective at evaluating and citing appropriate online resources within their written assignments.

## Introduction

### Designing Digital Literacy Education to Equip Learners to Evaluate Online Health Care Information Resources

Developments in online and digital media technologies are impacting the patient-health care relationship and creating a new area in which patients, health care students, and practitioners require guidance on how to operate. Although one can now access a wealth of health care information online, the lack of gatekeepers to review the quality of this information can contribute to the circulation of false information or misinformation in an online setting [1]. The availability of this misinformation can consequently contribute to misconceptions about issues in health care [1]. Misconceptions in this context can be defined as holding a view about a factual health care matter that is unsupported by scientific evidence and expert opinion [1,2]. These misconceptions can be particularly damaging in a health care setting when they alter individuals’ decisions to participate in evidence-based disease prevention or management strategies [1], for example, to opt out of vaccination programs or to eschew conventional treatments for complementary therapies [3,4].

The ability to critically evaluate the reliability and validity of online information is a shared component of the definitions of digital literacy and eHealth literacy (also known as digital health literacy) [5,6]. Defining the term digital literacy can be problematic, as it can encompass a range of computational skills on different digital devices and software [7]. Jisc defines digital literacies as, “capabilities which fit an individual for living, learning and working in a digital society”, providing a detailed framework for assessing digital literacy [5]. The capabilities that are encompassed in the Jisc definition are not health care context-dependent, and are tailored towards students in further or higher education [5]. eHealth literacy can be defined as the “ability to seek, find, understand, and appraise health information from electronic sources and apply the knowledge gained to addressing or solving a health problem” [6, p2]. The capabilities described in the definition of eHealth (digital health) literacy are tailored towards patients or members of the public [6,8]. However, there are several components common to both digital literacies and digital health literacies, including information literacy, ICT literacy, and online resource evaluation skills. Additionally, newer definitions of digital health literacy encompass online privacy skills, ensuring that the individual is capable of protecting their own and others’ privacy in an online setting [8]. Within the capabilities defined by digital literacy skills, learners are similarly taught to protect their own digital identity [5].

One of the challenges in the measurement of eHealth literacy is that metrics such as the eHealth literacy scale often rely on individuals self-reporting their perceived expertise [9,10]. Individuals often overestimate their perceived computer skills, and this may have contributed to the gap between perceived eHealth literacy and actual health literacy, as measured by computational performance tests [11]. Another challenge in the measurement of eHealth literacy is the rapid changes in the way health information is shared online. For example, the eHealth literacy scale was developed before social media and peer-to-peer sharing of resources became popular [12]. To adapt to these changes, later studies modified the eHealth literacy scale or developed novel digital health literacy scales [8,13].

Using these adapted scales, research has revealed that one of the capabilities that participants consistently feel least confident about is how to evaluate health care information online [8,13]. Interestingly, a study evaluating the views of health care professionals, in addition to patients and members of the public enrolled on the “Social Media in Healthcare” massive open online course (MOOC), showed that health care professionals also often found it challenging to evaluate health care information online [14]. Although learners felt confident about finding health care information online, over half of them were unsure about how to evaluate this information, particularly in the context of using this information to make health care decisions [14]. Owing to this, educational interventions to improve the ability of both patients and health care professionals to evaluate health care information online have been recommended [13,14]. However, many of these educational interventions aimed at improving digital health literacy skills have been taught as traditional classroom-based training sessions [15,16].

To date, little research has been conducted on online learning approaches aimed at improving the ability of learners to evaluate online health care resources. A recent study piloting e-learning on eHealth literacy in Japan found that 2 weeks of e-learning improved participants’ scores on both the eHealth literacy scale and on an assessment task, whereby students were asked to select which of the 5 websites they thought was most reliable, using a multiple-choice question [17]. These online educational activities were based on the learners’ reading materials on how to evaluate information, with several interactive multiple-choice questions to test understanding [17]. However, although the responses to multiple-choice questions permit rapid grading of individuals’ responses, they can lack authenticity and do not permit learners to explain their reasoning. More sophisticated tasks with open-text responses to determine whether individuals can demonstrate the ability to evaluate the information found online [18] can facilitate our understanding of learners’ reasoning. This in turn, can permit a more effective dialogue with learners, and the development of more effective educational interventions.

### The Broad Spectrum of Learner Stakeholders in Health Care Massive Open Online Courses

When designing educational interventions aimed at improving the ability to evaluate online health care resources, it is important to consider the different types of learners who may enroll in the program. In the context of a MOOC, there can be a broad spectrum of learners. In 2018, approximately 101 million learners enrolled in over 11,400 different MOOCs worldwide [19]. Courses on health and medicine comprised around 7% of the total, the equivalent of over 7 million learners [19]. A range of learner stakeholders who may benefit from participating in health care MOOCs has been identified [20,21]. These include (1) patients (and family members) who are seeking information about their condition, (2) members of the general public who are interested in improving their health literacy, (3) secondary school students who are considering applying for undergraduate health care degrees, (4) undergraduate students who may use MOOCs to revise or are encouraged to participate during their campus-based education, (5) health care professionals who are enrolled in MOOCs for the purposes for continuing medical education (CME) or continuing professional development (CPD), and (6) graduates who may be enrolling in MOOCs to enhance their curriculum vitae and considering postgraduate studies.

Owing to the heterogeneity in learner stakeholders, those designing health care MOOCs must plan for these to be accessible to a broad spectrum of learners, including patients, caregivers, and health care professionals [22]. Other MOOCs may be specifically designed to target a particular group of learner stakeholders, such as health care professionals, but these may envision reaching a smaller secondary audience of other groups of learners [23]. Table 1 highlights the potential range of learner stakeholders in health care MOOCs and the recent educational research studies that have evaluated the impact of these health care MOOCs.

**Table 1:** Learner stakeholders who may benefit from health care massive open online courses.

MOOC^a^ design	Learner stakeholders	Recent research studies
Patient education	Patients (and family members of patients) seeking information about their condition	Goldberg et al [22], Tieman [24]
Caregiver education	Caregivers for patients, who may not have completed any formal education on the patient’s condition	Goldberg et al [22]
Health literacy and public education	Members of the general public, who are interested in improving their health literacy	Atique et al [14] and Castle et al [25]
Outreach for secondary (high) school students	Secondary school students who are considering applying for undergraduate health care degrees	Stokes et al [26]
Integration into campus-based curricula for undergraduate students	Undergraduate students who may use MOOCs to revise or are encouraged to participate during their campus-based education	Swinnerton et al [20], Hossain et al [27], Robinson [28], and Jiang et al [29]
CME^b^ or CPD^c^	Health care professionals who are enrolled in MOOCs for CME or CPD purposes	Tribett et al [23], Fricton et al [30], Harvey et al [31], Magaña-Valladares et al [32], and Sarabia-Cobo et al [33]

^a^MOOC: massive open online course.

^b^CME: continuing medical education.

^c^CPD: continuing professional development.

### Considerations in Massive Open Online Course Instructional Design and Pedagogy

The MOOC platform FutureLearn has aimed to incorporate elements of Laurillard’s conversational framework in the design of the courses, to foster dialogue between learners as they progress through the course [34,35]. This framework promotes learning through discussion between the teacher and learner, as well as between the learner and other learners [34]. Laurillard proposes that there are 4 phases of the conversational framework: a *“discursive phase*” in which a teacher presents a new idea and discusses this with learners; “an *interactive phase*” where learners attempt the tasks set by the teacher and are provided with feedback; an “*adaptive phase*” in which learners begin to learn how to improve their application of key concepts as a result of feedback; and finally, a “*reflective phase*” in which learners reflect on the interactive and adaptive phases and may begin to articulate what they have learned [34]. The 6 different types of learning experiences within the conversational framework can be described as those based on acquisition, collaboration, discussion, investigation, practice, and production [36]. The different types of online media can support different aspects of the learning experience; these are summarized in Table 2, which is based on a previous work by Young and Perovic that maps FutureLearn MOOC activities to Laurillard’s 6 different types of learning experience [36,37]. By incorporating activities that elicit different learning types, learners gradually develop their understanding of a concept, begin to apply their understanding in a scaffolded environment, and then progress to more complex activities either individually or in small groups.

**Table 2:** Categorization of massive open online course media activity by learning experience.

Learning experience	Description	MOOC^a^ media activity
Acquisition	Learners are introduced to a concept or learn more about a concept, but are not asked to undertake any action or articulate their understanding	Video, article, and podcast
Discussion	A stimulus for discussion is generated for learners to discuss their emerging understanding of the concepts	Online discussion forums, online hangouts with educators, and Twitter chats
Inquiry	Learners are guided in their search for additional resources to build upon their understanding of the concept	Web search, database search, case-based learning, and problem-based learning
Practice	The learners undertake activities that allows the learners to apply their understanding of the concept and receive feedback on their work	Virtual learning environments, programming tasks, and assessments such as automated multiple-choice tests with feedback
Collaboration	Learners are tasked with creating a joint product and expected to articulate their decision-making process to other learners throughout this activity	Small group projects, online discussion forums, and creating Wikipedia pages
Production	The learners are asked to generate a piece of work that allows the learners to articulate their understanding of the concept	Creation of digital files (video, podcast), written assignments, peer reviewing assignments, writing new code, webpage, and blogs

^a^MOOC: massive open online course.

### The Aim of the Study

The health care MOOC studied here was designed to be accessible to a broad spectrum of learners, such as patients, caregivers, students, and health care professionals. The aim of this study was to evaluate a series of educational interventions that were aimed at improving the ability of individuals to evaluate online health care information. This research focused on finding out whether any of the educational interventions were successful and, if so, examining the reasons for their success. Data from 8 different “runs” of the MOOC were collected. In 4 of the 8 runs, changes were made to the learning design of the MOOC, with the aim of improving digital literacy education. To evaluate the impact of these interventions, data on learner performance in and their comments around these educational interventions were collected. Data were analyzed using a mixed methods approach. Metrics such as the inclusion and appropriate citation of resources in the summary assignment were taken as a proxy for the successful evaluation of online resources. This study, on a course that has run 8 times over a period of 5 years, answers the call for longitudinal studies on the impact of educational interventions and their iterative refinement in MOOCs [38,39].

## Methods

### Ethical Considerations

Ethical approval for the educational research in this study was obtained from the MVLS College Ethics Committee at the University of Glasgow. This study was conducted in accordance with the Research Ethics for FutureLearn guidelines [40].

### Background

Data were gathered from 8 separate runs of a 6-week MOOC, “Cancer in the 21st Century: The Genomic Revolution.” These 8 separate runs of the MOOC took place between May 2014 and February 2019 on the FutureLearn platform. This MOOC contains a brief written summary assignment (300 words), which was peer reviewed by other learners enrolled in the course. Both the summary assignment and peer review were scheduled in the final (sixth) week of the course. The assignment topic was epigenetics and cancer; the assignment question was, “What do we know about how epigenetic regulation goes wrong in cancer and what types of targeted treatment could arise from our knowledge of epigenetic deregulation in cancer?” Learners were asked to list the resources they had used at the end of the summary and were advised that this reference resource list was not included in the indicative 300-word limit for the assignment. A total of 4 open-access papers were set and given as optional reading for this assignment; learners were also encouraged to identify their own resources to include.

The baseline guidance provided in all runs was as follows: (1) a short video and a short article on the topic of epigenetics in cancer introduced learners to the assessment topic. (2) a total of 2 short videos were created by a College librarian, specifically for the learners in this course, 1 video on how to conduct searches online for resources (“Getting the Most Out of Google”) and 1 video introducing learners to the freely accessible PubMed database, “Using a Scientific Literature Database.”

### The Written Summary Formative Assessment and Iterative Changes to Guidance

The series of iterative changes and the learning types classification of the media involved in the changes to the learning design of the MOOC are illustrated in Table 3. Within Table 3, the iterative changes to the digital literacy guidance and preassessment digital literacy tasks are outlined below, indicating in which “‘Run”’ of the course these changes were introduced. The learning type that these activities were designed to elicit are also described; these are either acquisition, collaboration, discussion, investigation, practice, or production [36].

**Table 3:** Iterative changes to the written summary assessment guidance and preassignment tasks.

Run	Date	Iterative changes	Learning type goal
1	May 2014	Baseline: Learners were shown short videos on searching for information online called, “Getting the Most Out of Google” and “Using a Scientific Database”; Learners were guided in their search online to find information about a specific type of cancer; Learners were provided with a written brief on assignment content and asked to list their references at the end of the assignment; Learners were also asked to review their peers’ assignments and to provide written feedback	Acquisition: short videos on how to find online resources; Collaboration: discussion of how to approach the search for resources; Inquiry: students were asked to conduct their own Web/database search to find additional information and then post this to a discussion forum; Production; write a short-written assignment and peer review other learners’ assignments using a rubric
2	August 2015	Additional plagiarism check and assessment guidance were briefly provided in written form. A link to a detailed webpage providing information on plagiarism was added to the assessment briefing	Acquisition: additional written information on plagiarism and assessment guidance
3	January 2016	The written plagiarism guidance was expanded and edited to improve clarity and succinctness. Students were no longer directed to a long, detailed webpage (likely unsuitable for those new to concept of plagiarism)	Acquisition: modified written information on plagiarism and assessment guidance
4	April 2016	No changes	N/A^a^
5	January 2017	No changes	N/A
6	September 2017	No changes. (nb from this run onward; only learners who had paid for a certification option could complete the assessment)	N/A
7	January 2018	An additional preassessment digital literacy task was included on “Links between environmental agents and cancer: how to find reliable information”; Learners were asked to evaluate which of the 4 websites was the most reliable source of information and enter their answer in a poll; Learners could further discuss why they selected a certain website in the discussion forum for this poll; The assessment briefing was modified to include a short citation guidance, and it included links to additional webpages for further reading	Practice: students could apply their understanding of the concepts surrounding resource evaluation; Discussion: learners could further discuss their rationale for resource evaluation
8	January 2019	Additional preassignment digital literacy guidance was added to this run. A new 3-step section of the course was created, called “Evaluating sources on the internet: can you believe what you read about cancer?” This section featured 3 short videos: “Source Evaluation: Author and Organization,” “Source Evaluation: Website Content,” and “Source Evaluation: Summary”	Acquisition: learners received more advanced information on how to evaluate online resources; Discussion: each video has a discussion forum for learners to discuss the concepts in each video further

^a^Not applicable.

### Guidance for Learners on Conducting Peer Reviews of the Written Summaries

After they had submitted their written summary assignment, learners were asked to review their peers’ written summaries by answering the following questions:

1. What did you like about the author’s work?
2. Had the author carried out research using reliable resources and had good use been made of these?
3. How might the author improve the communication of their key ideas?

There was no limit on the number of peer reviews that learners could write for their peers.

### Calculating the Similarity Index in Massive Open Online Course Assignments

Following the first run of the MOOC, a similarity index for each of the submitted summary assignments was calculated by submitting all 203 learner summary assignments to the plagiarism-detection software Turnitin. A high Turnitin similarity index indicates potential plagiarism. “Summary nonsubmission” assignments were defined as those that included no written summary, that is, they were submitted as a “dummy” assignment [41]. The 32 summary nonsubmission assignments were removed from the analysis, and the remaining 171 summary assignments were categorized into 5 separate tiers based on the Turnitin similarity index: “no matches,” “1 matching word to 24% similarity,” “25% to 49% similarity,” “50% to 74% similarity,” and “75% to 100% similarity” [42]. The number of words in the areas of text in each written summary, which were highlighted as having similarity with other texts by Turnitin, was then divided by the total word count for that summary (excluding the resource list). Assignments in Runs 2 to 7 of the program were not submitted to Turnitin to determine the similarity index, as consent for this had not specifically been sought from participants in these runs of the MOOC.

### Analysis of Massive Open Online Course Assignments and Peer Reviews

MOOC summary assignments were manually analyzed to evaluate the number of sources listed at the end of the summary, and an average and SD were calculated for each “run” of the course. The differences in the number of references listed per summary assignment in each run were compared using a two-tailed Student *t* test.

In addition, the summaries were manually coded into 3 groups: “includes a list of sources”; “learner writes that the recommended resources were used”; “no sources listed or no reference to sources.” For summaries to be categorized as “includes a list of sources,” learners may have included a conventional reference list or a list of weblinks to the articles or websites used as resources; academic citation formats were not required. The proportion of each group in each “run” of the MOOC was calculated as a percentage to enable comparisons across runs. Fisher exact test was used to calculate whether the numbers of each type of assignment were significantly different between successive runs.

### Evaluation of the Preassessment Digital Literacy Tasks

For the preassessment digital literacy poll task (Runs 7 and 8), the learners were asked to review 4 different online resources that provide information on links between wearing underwire bras and cancer. A quantitative analysis of which source learners selected to be most reliable out of the 4 sources provided was performed. A qualitative analysis was performed on the learners’ comments that related to why they chose those particular resources. These included comments on the poll activity, subsequent discussion step, and “Getting the Most Out of Google” video step that aimed to improve the learners’ digital literacy skills.

## Results

### The Number of Learners Who Submitted Summary Assignments in Each Run

Table 4 shows that the total number of active learners follows a general downward trend over Runs 1 to 8. Active learners are defined by FutureLearn as learners who mark at least one step on the course as “‘complete.” Similarly, in Run 1, 171 learners submitted summary assignments, and by Run 8, this number had dropped to 17 learners submitting assignments. The total number of summary assignments submitted, presented in Table 4, exclude summary nonsubmissions. In Run 6, a change in the mode of certification was introduced by FutureLearn: only learners who paid for a certificate of completion were able to access the written summary assignment and peer-review task. This likely contributed to the decrease in the number of learners completing the summary assignments. In Run 5, 76 learners submitted a summary assignment, and 145 peer reviews were written in total. In Run 6, with the new payment model, this dropped to 15 learners submitting a summary assignment and a total of 22 peer reviews. In Run 8, 17 written summaries were submitted, and a total of 46 peer reviews were written. The number of peer reviews written, shown in Table 4, include peer reviews written about summary nonsubmission (“dummy”) assignments, as these were qualitatively reviewed to evaluate the learner response.

**Table 4:** Summary of the number of active learners, learners who completed over half the course, assignments submitted, and peer reviews completed for each massive open online course run.

Run	Active learners	Completed >50%	Summary assignments submitted	Peer reviews written	Peer reviews per summary assignment	Learners who marked the poll step as complete
1 (May 2014)	2153	757	171	327	1.9	N/A^a^
2 (Aug 2015)	2564	623	100	149	1.5	N/A
3 (Jan 2016)	2329	578	102	137	1.3	N/A
4 (Apr 2016)	1606	311	48	69	1.4	N/A
5 (Jan 2017)	1811	450	76	145	1.9	N/A
6 (Sep 2017)	676	147	15	22	1.5	N/A
7 (Jan 2018)	789	175	14	19	1.4	176
8 (Jan 2019)	610	151	17	46	2.7	182

^a^Not applicable.

### An Analysis of the Levels of Plagiarism in the Written Assignments in the First Run of the Massive Open Online Course

Table 5 shows the levels of matching text within the 171 written assignments submitted by learners in Run 1 of the MOOC and previously published sources. The percentage similarity index calculates the total number of words in the text that match previously published sources or other student work in the Turnitin database (excluding any references or resource lists). Although a majority of the assignments have relatively low levels of text that match previously published sources, 15.2% (26/171) of the assignments have a similarity index over 50% (11/171, 6.4% of the assignments are in the 50%-74% similarity index category and 15/171, 8.8% of the assignments are in the 75%-100% similarity index category). Although similarity indices could not be obtained for Runs 2 to 8, we observed that at least one and as many as 5 assignments from each run were fully plagiarized from either the Abstract or Introduction section from a previously published review article on the topic. In many instances, the review articles that were plagiarized were on the recommended reading list for the assignment. Of note, a particular review article titled “Epigenetics in Cancer [43],” which was not on the assignment reading list, was plagiarized in the summary assignments submitted in Runs 3, 4, 5, 6, and 8 of the MOOC. This is an open-access article, and a link to this article is one of the first results to appear in a Google search for the terms “epigenetics” and “cancer.”

**Table 5:** The evaluation of plagiarism in the learner summary assignments in Run 1 of the course.

Similarity index of assignments		Percentage of assignments (N=171), n (%)
No matches		16 (9.4)
**Assignments with matching text**		
	One matching word to 24%	101 (59.1)
	25%-49%	28 (16.4)
	50%-74%	11 (6.4)
	75%-100%	15 (8.8)

### Learners’ Reactions to Their Peers’ Written Work in Run 1

During the peer-review process, learner peer reviewers were asked to answer 3 questions: “What did you like about the assignment?”; “Did they make good use of resources and was the assignment well referenced?”; and “How could they make improvements to the assignment?” During this analysis, peer reviews of summary nonsubmission (or “dummy”) assignments or illegible peer reviews were excluded. This reduced the total number of peer reviews from 327 to 264. When answering “Had they carried out research using reliable resources and had good use been made of these?,” 67 out of 264 peer reviews (25.4%) mentioned that there were no references in the assignment. In answering “How might the author improve the communication of their key ideas?,” 29 out of 264 peer reviews (10.9%) mentioned that the assessments could be improved by more accurate or appropriate referencing. These comments were overwhelmingly positive, with peer reviewers commenting that the learners must have used references because of the quality of the assignment, as well as mentioning that that they were not listed, for example, “There are no references, but it reads as if you have researched well and used various sources of information.” In certain cases, learners went out of their way to defend their peers’ lack of references in the assignment, citing the 300-word limit on the assignment as a possible reason, for example, “I am not sure what the sources were but as when I was doing my submission it is difficult to reference with the small word count.”

In contrast, where learners identified plagiarism in their peers’ assignments, their review comments seemed to indicate less tolerance of this transgression compared with their response to learners omitting references, as reviewers did not minimize or excuse apparent plagiarism. A total of 2 learners noticed high levels of plagiarism in the assignments that they had been allocated to peer review; their responses are shown in Table 6.

When asked to comment on the written assessment, a learner stated that they themselves were too pressed for time to undertake a written assessment and subsequent peer review: “Unless I resort to outright plagiarism I do not have time for this exercise…Whether you like the author’s style or communications ability is not an issue and how one can evaluate the use of resources beats me.”

**Table 6:** Summary of learners’ responses to their peers’ plagiarized assignments in Run 1. The Learner Reviewer ID and the Assignment ID have been renamed to ensure anonymity. The 3 questions used to scaffold the peer reviews are shown alongside the learners’ answers to these questions.

Learner reviewer ID	Assignment ID	What did you like about the author’s work?	Had the author carried out research using reliable resources and had good use been made of these?	How might the author improve the communication of their key ideas?
1	X	I cannot judge the author's work, as the work is mainly a copy of an abstract from an article	The source was reliable, peer reviewed, but the work is mainly a copy of the abstract of the paper	Not to copy: need to write an original piece answering the questions asked for the assignment
2	Y	It relates to real work in the lab	No, in fact, large chunks were lifted verbatim from here (The webpage of the Cancer Epigenetics Lab at the University of Bristol); They also did not explain what epigenetics was.	They need to answer the question

### Evaluation of the Number of References in Learner Summary Assignments

In the assessment guidance, the learners were asked to list the resources that they used to write their assignment at the end of the summary. The average number of references per written assignment was calculated (Table 7). The introduction of additional referencing and plagiarism guidance in Run 2 did not significantly increase the average number of references per summary: the average number of references for Runs 1 to 6 ranged between 1.8 and 2.4 references per summary. The introduction of additional preassignment digital literacy tasks in Runs 7 and 8 increased the average number of references per summary to 2.9 and 3.5.

**Table 7:** The average number of references per final summary assignment.

Run	Average number of references per summary, mean (SD)	*P* value from *t* test (*df*) comparing the difference between the mean in Run 1 with the means in Runs 7 and 8
1	1.8 (1.9)	N/A^a^
2	2.1 (2.3)	N/A
3	2.4 (2.7)	N/A
4	2.2 (2.5)	N/A
5	1.7 (2.1)	N/A
6	2.0 (2.0)	N/A
7	2.9 (1.8)	.049 (183)
8	3.5 (2.9)	.001 (186)

^a^Not applicable.

### An Analysis of the Different Types of Referencing in Learner Summary Assignments

**Figure 1:**
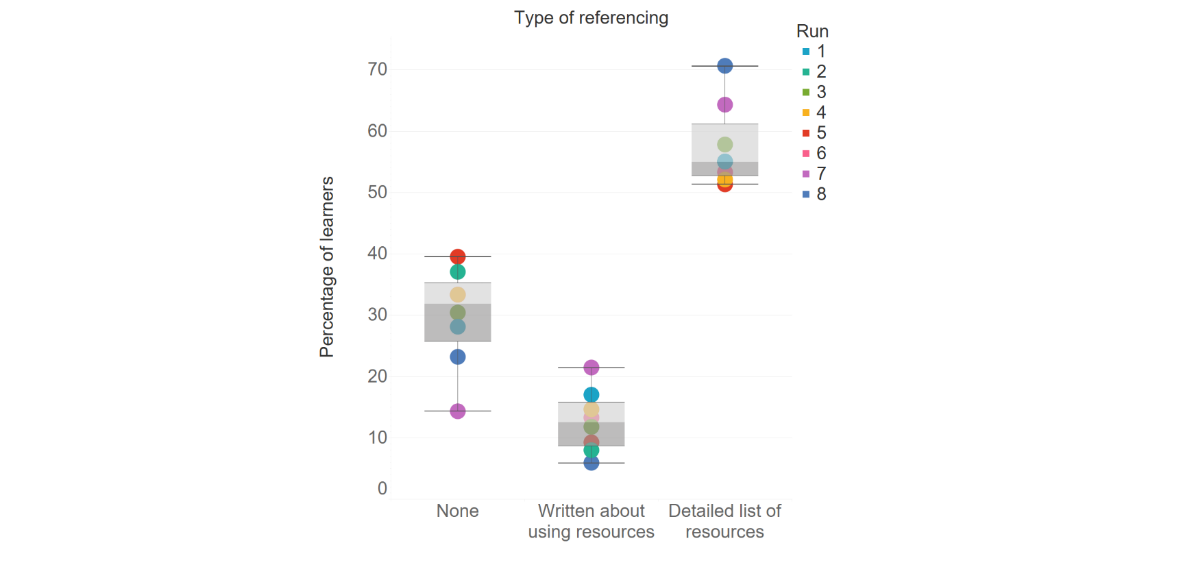
Analysis of the type of referencing in learner summary assignments.

To evaluate the trends in the types of referencing adopted by learners, written summaries were manually coded into 3 categories: “no references listed”; “wrote about using resources”; and “detailed list of resources.” Upon finding that around 15.2% (26/171) of the learner assignments in Run 1 contained a Turnitin similarity index score of between 50% and 100%, new referencing and plagiarism guidance was added to the written summary assignment guidance in Run 2. Figure 1 shows that the initial introduction of plagiarism guidance in Run 2 did not seem to affect the percentage of learners listing references in their written summaries (Run 1: 55% and Run 2: 55%). In contrast, the addition of a preassessment digital literacy task in Run 7 increased the percentage of learners who included a reference to 64%. After adding an additional guidance video on evaluating online resources, as well as an additional preassessment task in Run 8, the percentage again increased to 71%.

In Runs 1 to 6, the percentage of learners who submitted a summary with neither a list of resources nor write-ups about their resources in the summary ranged between 28% and 40%. In Run 1, this percentage was 28%, and following the introduction of new referencing and plagiarism guidance in Run 2, this level did not decrease (Run 2: 30%). In Run 7, following the addition of a preassessment digital literacy task, the percentage of learners who submitted a summary without a list of resources or writing about their resources in the summary was reduced to 14%. Throughout all runs, a smaller subset of learners (between 6% and 21%) obliquely wrote about using resources in their summary, such as those listed as recommended reading, but they did not provide a reference list at the end of the assignment.

**Figure 2:**
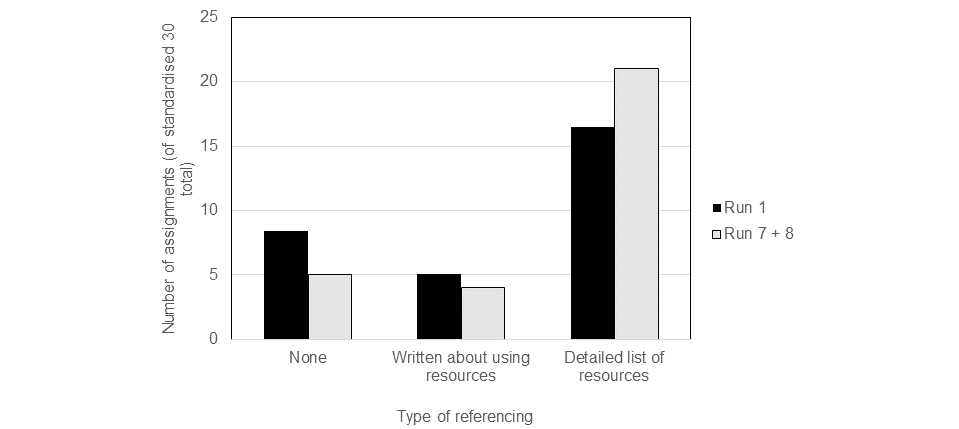
Change in the predominant type of referencing in learner summary assignments between Run 1 and Runs 7/8.

Figure 2 shows the number of assignments in each referencing category, Run 1 compared with Runs 7 and 8. A representation of the distribution of types of summaries is shown in black bars in Figure 2. The total number of summary assignments in Runs 7 and 8 have been combined and are represented by the grey bars. Run 1 (black bars) had the least guidance and Runs 7 and 8 (grey bars) the most digital literacy guidance. Note that for the graph in Figure 2, because of the far greater total number of assignments in Run 1 than later runs, the total assignment numbers in Run 1 were normalized before comparison with Run 7/8 numbers, by converting them to a number out of 30 (the total number of Run 7/8 assignments). A one-tailed Fisher exact test was used to calculate whether the increased number of well-referenced assignments was significantly greater in Runs 7/8 than in Run 1. Although the difference in the proportion of assignments of each type did not meet statistical significance between Run 1 and Run 7/8 (*P*=.11), this was partly because of the small number of assignments in the later runs. Nonetheless, there was a clear trend toward improved referencing in Runs 7 and 8 compared with Run 1 (Figures 1 and 2).

### A Qualitative Analysis of Learner Comments

Overall, the learner poll results and comments suggest beneficial effects of the preassessment tasks and digital literacy skill guidance. Most learner comments (12 out of 16 learner comments) on the practice poll activity in Runs 7 and 8 demonstrated correct evaluation of resource reliability, with appropriate justification(s). Furthermore, some comments suggested additional valid factors (not included in the MOOC teaching) that could be used to consider when assessing reliability, such as whether the source is associated with marketing products for sale or population used in a scientific article was representative of the general population. The remaining comments indicated uncertainty (2) or incorrect conclusions (2) about resource reliability, suggesting that the majority of learners possessed good awareness of this topic at the end of this step. Similarly, although there were a few (7) comments specifically relating to the source evaluation videos (Run 8 only), these were all positive. Strikingly, the “Getting the Most Out of Google” video, which detailed how to use advanced Google search functions, was one of the MOOC’s most popular videos, with a total of 857 positive learner comments out of 909 comments across all runs, with most of the remainder either neutral or unrelated to the video content.

## Discussion

### Learning Design Considerations for Online Educational Interventions Aimed at Improving Digital Health Literacy Skills

The demographic analysis reveals that a range of learner stakeholders, including learners pursuing higher education, patients, and family members of those with cancer, along with current and past health care professionals, was enrolled on this health care MOOC. Previous studies have identified a common learning requirement for these groups of stakeholders: the critical evaluation of the reliability and validity of online resources that contain health care information [8,14]. By including a series of tasks aimed at improving these skills, learners engaged in more in-depth conversations on how best to evaluate online resources and included a greater number of appropriate citations in their written assignments. It could be argued that these tasks improved the ability of the learners to critically evaluate a range of online resources with varied value [44].

The nature of this MOOC, run 8 times over a period of 5 years, permitted an iterative approach for the educational interventions aimed at improving digital health literacy skills. The early runs of the course indicated that the base-level educational interventions did not fully support all the learners in developing the skills for evaluating online health care resources. Over 15.2% (26/171) of the written assignments contained plagiarized text, which may indicate a superficial engagement with the online resources. Furthermore, learner comments in a reflective task indicated that the learners did not feel confident in evaluating online resources.

Following a review of the MOOC’s content, which was aimed at improving the learners’ ability to evaluate online health care information, a gap in “practice” or scaffolding activities for learners was identified [36]. These are activities in which the learners can begin to apply their learning on clearly defined tasks, with formative feedback from their peers or educators enrolled in the course [22]. When scaffolding tasks were included in the later runs of the course, within a sequence of activities aimed at teaching learners how to evaluate online resources, a significant increase in the average number of appropriate citations per written summary was found. These scaffolding activities included a task in which learners were asked to vote in a poll for which resource they thought was the most reliable, view the overall poll results, and comment on their choices. Finally, in Run 8, 3 new videos were added to the course to aid learners in the evaluation of online resources. The learners’ discussion about these new activities in their associated discussion forums suggested beneficial effects of these activities, including improved understanding of concepts, such as source reliability and validity in the evaluation of online resources. Learner comments also suggested useful points that could be incorporated into future tasks and guidance on how to evaluate online health care information.

A recent study aimed at improving digital health literacy taught learners by using activities that elicit 2 learning styles: “acquisition” and “practice” [17,36]. Mitsuhashi found that this improved the self-reported digital health literacy scores of the learners who completed the educational program [17]. In this study, we suggest that a range of activities designed to elicit different learning styles [36] was key in the development of the online resource evaluation skills for this diverse set of learner stakeholders. The activities aimed at improving the evaluation of online resources were designed to elicit 5 different learning styles (examples shown in Multimedia Appendix 1): “acquisition,” “discussion,” “inquiry,” “practice,” and “production.” The addition of the short “practice” poll activity in which learners were asked to evaluate a range of resources was much more popular than the summary assessment (production), with approximately 10 times as many learners completing this assessment. We cannot draw conclusions about engagement with these activities here; however, learners may perceive the written summary assignment to be too time consuming (as indicated by learner comments), and the introduction of a fee in the later runs may have specifically reduced engagement with the written assignment. We suggest that a learning design approach that includes both written and poll-based tasks may engage a wider proportion of learners. A key finding is that, for the small group of learners who completed the written assignment, these combined interventions led to an increase in the number of appropriate citations. This finding has particular relevance in the context of peer-to-peer sharing of online sources of health care information: individuals who cite appropriate resources may be more successful in combating health care misinformation shared online in a peer-to-peer social media context [1]. Improving the learners’ ability to appropriately evaluate online health care information may also improve their ability to combat health care misinformation (eg, social media) [1].

### Plagiarism and Professional Identity: The Requirements for Digital Literacy Guidance in Health Care Massive Open Online Courses

In addition to teaching learners about the evaluation of online resources, another approach to support learners in developing their digital literacy skills in an online setting may be to encourage them to consider how their behavior online may impact their professional identity. “Career and identity management” is another element of digital literacy, which is defined by Jisc [5]. This is of particular importance for learner stakeholders, such as undergraduate students training for a professional degree and health care professionals. Macfarlane writes that “what it means to be a student, not just the product of their intellectual endeavours undertaken in private, is now observed and evaluated” [45]. These concerns may be amplified in the large-scale MOOC setting. Many MOOC platforms encourage learners to enroll with their real names, and comments made on discussion forums are widely available. In such a public online setting, undergraduates enrolled in professional health care degree programs (similar to teacher training and social care training) are subject to additional scrutiny because of the expectations surrounding professional behavior. Professional bodies that regulate degree accreditation have begun to increasingly include expectations for practitioners in an online setting, including social media [46,47]. In Run 1 of this course, over 15.2% (26/171) of written summary assignments contained plagiarized text. However, the penalties for plagiarism in health care MOOC assignments are unclear, and plagiarism guidance is often absent. These findings highlight that to prepare learners for written assessments in health care MOOCs, guidance on digital literacy, in relation to career and identity management, should be provided.

This is of particular relevance for any written assignment in a MOOC taken by health care professionals for CPD or CME purposes. In addition, these findings highlight the potential benefit of an “in-house” discussion on appropriate professional conduct in an online setting, for health care learners who are advised to study MOOCs to supplement their learning during their undergraduate or postgraduate degrees. Including additional guidance on good conduct within open online courses and social media platforms may aid the development of learners’ career and identity management digital literacy skills [5].

### Limitations and Future Directions

Although most of the educational activities described above were freely accessible to all learners, the written peer assessment task was not: from Run 6 onward, learners had to pay for a certificate to access written assessments. This may have resulted in the selection of a subset of learners who were highly motivated to engage with and successfully complete the peer assessment task analyzed in this paper. The findings from this written assessment task may therefore not be fully representative of all learner stakeholders in this MOOC. Concerns regarding the analysis of learning analytics data from small subgroups or small “samples” of the learner population in MOOCs have been raised previously [48]. In a MOOC setting, the risk of assuming that data from a small subset or sample of learners represent the wider population of learners may be amplified [48]. Learning analytics based on a small subset of MOOC learners may skew our understanding of how learners develop skills in the evaluation of online health care resources [48]. Caution is warranted in the interpretation of findings from a small group of learners, particularly when there is a high level of heterogeneity in learner stakeholders who have different motivations, as well as educational and professional backgrounds. However, the number of summary assignments and peer reviews submitted in each run reflected the total number of active learners enrolled in the course in that run. This suggests that participation in this formative assessment task is likely to reflect the overall levels of learner engagement, both before and after the introduction of the paywall that restricted access to the assessment tasks.

A quantitative comparison of the similarity index across all the runs of the MOOC would enable a statistical analysis of the impact of the introduction of the range of educational interventions on the levels of plagiarism in learner assignments. Owing to limitations in consent from learners for this specific analysis in Runs 2 to 8, only a qualitative estimate was performed. Although our findings suggest improved referencing following the iterative educational interventions in the later runs, it would be beneficial to carry out a direct test of implementing all of our digital literacy interventions at once in a health care MOOC that currently lacks such guidance. Such a direct test could be carried out in a MOOC with large learner numbers. This is needed to confirm our finding that increasing digital literacy guidance and tasks correlated with an increased number of assignments containing a description of the resources used.

To support the diverse range of learner stakeholders enrolled in the MOOC, with varying subject-specific expertise and educational backgrounds, the inclusion of assessment exemplars was avoided. Exemplars might have been interpreted as proscriptive by learners, and a wide variety of exemplars would have been needed to cover the likely writing styles and levels of the diverse learner groups. Nonetheless, in future runs, a range of exemplars could be included to showcase a range of assignments with the appropriate use and acknowledgment of online resources, despite varying content and writing style.

Finally, because of enhanced learner data protection regulations, we could not link the learners’ information provided in surveys to their discussion of the digital literacy guidance, assessment, or the peer-review exercise. We were therefore unable to determine whether particular demographic categories of learner stakeholders, such as previous education level, influenced the learners’ experience of or learning gain from these MOOC activities. Although this digital literacy training appeared to benefit all learner stakeholders, a more detailed analysis of how the different learner stakeholder groups engaged with these activities would be highly informative for the design of future health care MOOCs to promote digital health literacy.

### Conclusions

This study demonstrates how a series of digital health literacy educational activities can be incorporated successfully in a health care MOOC and provides a possible blueprint for future online educational interventions aimed at improving digital health literacies. Importantly, this study shows that these educational interventions were most successful when the learning requirements of all the learner stakeholders enrolled in the MOOC were considered. The final and most successful guidance and preparatory steps were tasks that scaffolded learners in the critical evaluation of online health care information. We suggest that this approach is applicable to a wide range of online courses, such as health care MOOCs, that have a diverse range of learner stakeholders, including students preparing for undergraduate professional health care degrees, health care professionals, patients and their families, and professionals working for health care charities.

## Appendix

Multimedia Appendix 1 The final learning design of the digital literacy educational interventions aimed at improving the ability of learners to evaluate online health care resources. A description of each of the different learning activities and learning types are provided.

## Abbreviations

CME: continuing medical education
CPD: continuing professional development
eHealth: electronic health
MOOC: massive open online course
