# Supplementary material for: Equipping Learners to Evaluate Online Health Care Resources: Longitudinal Study of Learning Design Strategies in a Health Care Massive Open Online Course
Source: J Med Internet Res. 2020 Feb 26;22(2):e15177. doi: 10.2196/15177 (PMC7066506; doi:10.2196/15177)
Supplement: Multimedia Appendix 1 [file jmir_v22i2e15177_app1.pdf]

Multimedia Appendix – Final Learning Design of Digital Literacy Educational Interventions

Week 1

Learning activity – video and discussion forum on how to conduct an online search using Google.

Learning types – acquisition (video), learners are shown how to conduct searches online using the Google search engine.

Discussion (discussion forum associated with video), learners discuss the concepts explained in the video.

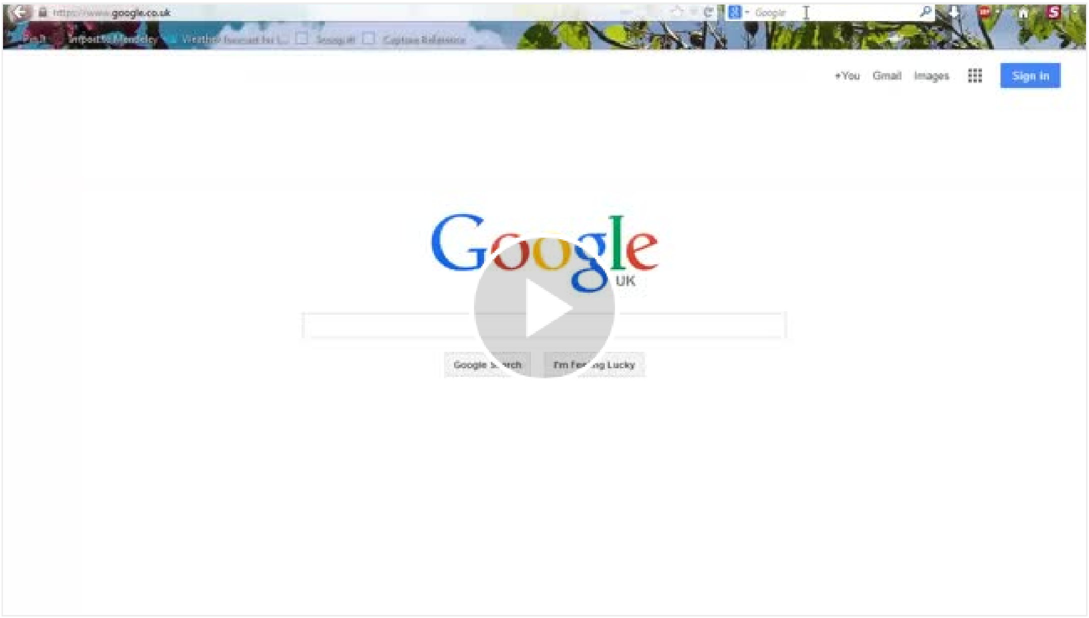

View transcript | Download video: standard

Getting the most out of Google

43 comments

At various points throughout this course we're going to be getting you to do some investigating. So we've asked one of the librarians at the University of Glasgow to give you some advice on how to get the most out of internet-based search engines.

Week 3

Learning activity – guided online search activity to find out more information about cancer statistics.

Learning type – inquiry, students are asked to conduct their own search outwith the massive open online course platform. They are guided on the search topic and are also provided with suggested search locations in the form of online resources.

Your turn: Investigating cancer statistics

52 comments

Now that you've listened to Professor Hanlon talking about how we examine cancer statistics we want you to do some investigating of your own.

Pick one type of cancer - maybe one that you feel you don't know much about and investigate some of the following questions. Don't feel you have to answer them all - they are just a guide to help you do some of your own research.

What is the incidence of the cancer? Is this the same worldwide?

Have there been any changes in incidence noted in recent times?

What is the prognosis for the cancer as a whole?

Is this different depending on particular features or classifications of the cancers?

Are there any risk factors known and how were these established?

A couple of good starting points might be:

http://seer.cancer.gov/statfacts/

http://www.cancerresearchuk.org/

Share your findings here. Please also read your fellow learners' comments. You can reply to others' comments, and 'like' comments that you find particularly interesting or agree with. Remember you can also 'follow' other learners, and filter comments by the number of 'likes' they have. You can also see any replies to your comments in your activity feed.

© Dr Leah Marks, University of Glasgow

Learning activity – poll and discussion on the evaluation of online resources.

Learning types – practice (poll), learners are presented with links to four online sources to evaluate and are then asked to vote on the online source they consider to be the most reliable.

Discussion (discussion forum associated with forum) - learners articulate why they considered a particular resource to be the most reliable.

Links between environmental agents and cancer: how to find reliable information

8 comments

In the next step, we will ask you to choose a particular cancer that you are interested in, and find out about any possible links between environmental factors and that cancer. In preparation for this, in the current step, we would like you to think about how reliable the information you find online is.

You might want to consider: are the websites, books or videos that you got information from, written by people who are biased in any way? Are they written by qualified experts in the subject area? Is the evidence they present convincing? Is it up-to-date?

To help you think about this, we have posted four links below, to websites that all have information about whether wearing underwire bras can cause cancer. Please have a look at each of the four websites that have written about the potential connection between wearing underwire bras and cancer, and then answer the Poll below:

Link 1: GOOP: bras and breast cancer.

Link 2: Stop Cancer Fund: bras and breast cancer

Link 3: Brafree.org

Link 4: CEBP Journal

Which of the four online articles did you think was the most reliable?

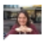

Leah Marks LEAD EDUCATOR

Please post some comments about how you decided on which website was the most reliable. What factors did you consider? Which did you think was least reliable, and why?

Learning activity – video and discussion forum on how to evaluate online resources by checking information about the author and the organisation that publishes the online content.

Learning types – acquisition (video) and discussion (discussion forum associated with video).

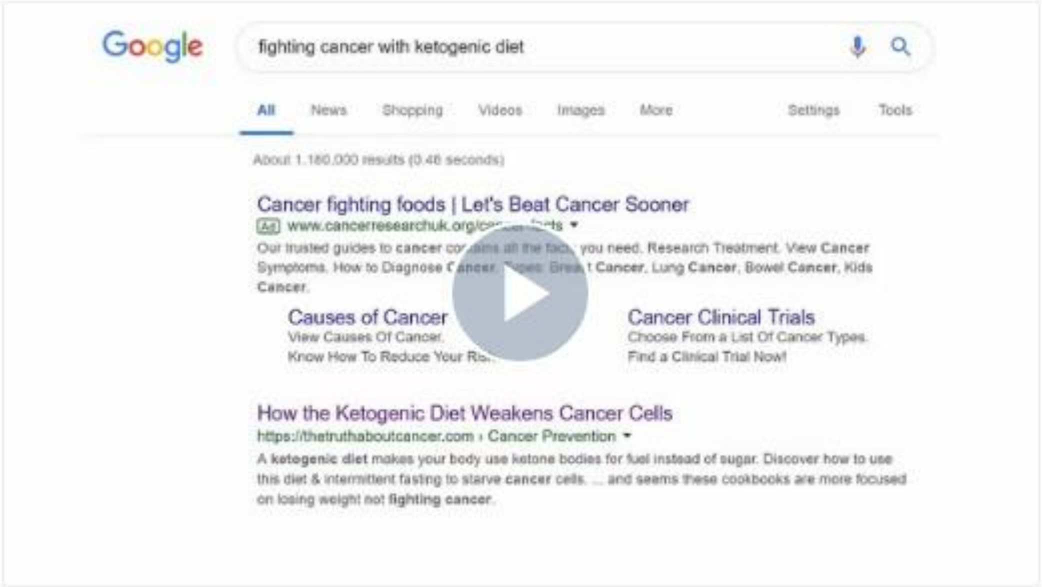

View transcript

Source evaluation: author and organisation

7 comments

© University of Glasgow

Learning activity – video and discussion forum on how to evaluate online resources by checking information about the content of the website. This involves evaluating the resources cited by the website independently and conducting one's own online search to find out if this matches the information presented.

Learning types – acquisition (video) and discussion (discussion forum associated with video).

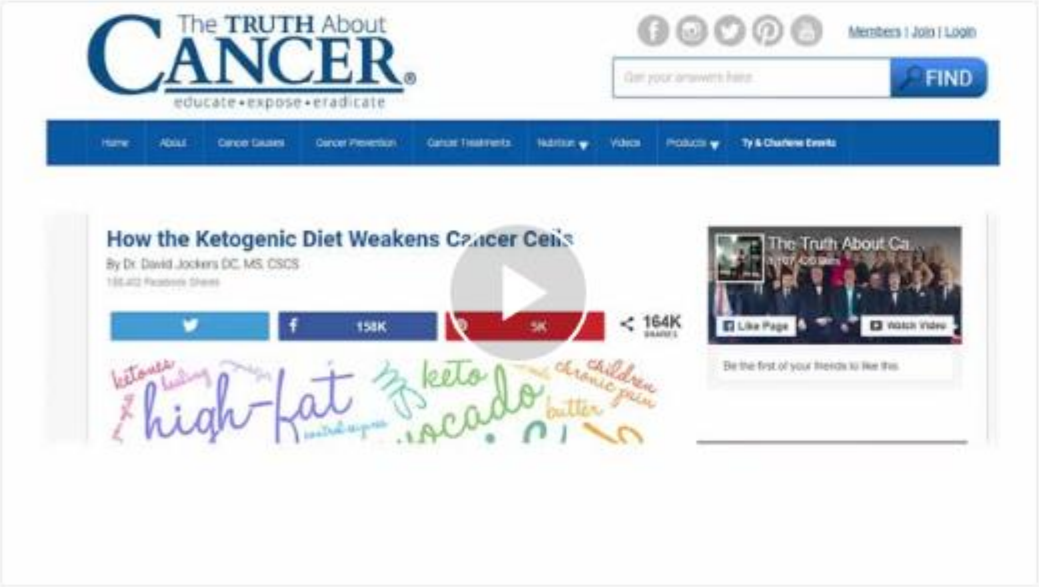

View transcript

Source evaluation: website content

3 comments

© University of Glasgow 2018

Learning activity – video and discussion forum summarising the concepts presented on how to evaluate online resources.

Learning types – acquisition (video) and discussion (discussion forum associated with video).

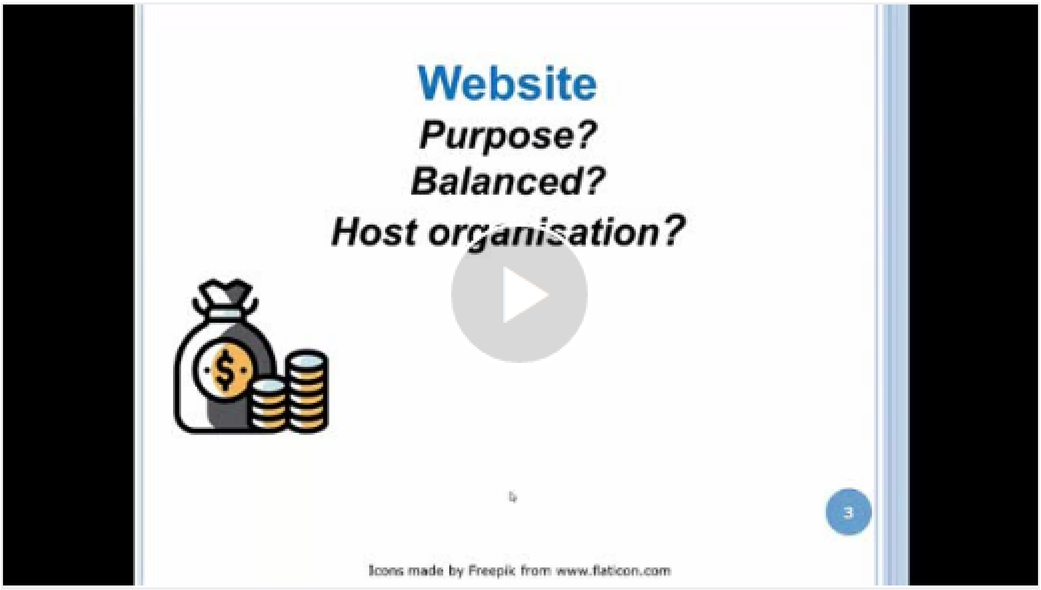

View transcript

Source evaluation: summary

Week 6

Learning activity – video and discussion forum on how to conduct an online search using the PubMed database.

Learning types – acquisition (video), learners are shown how to conduct searches online using the PubMed database (a database maintained by the United States National Library of Medicine at the National Institutes of Health).

Discussion (discussion forum associated with video) where learners chat about the concepts explained in the video.

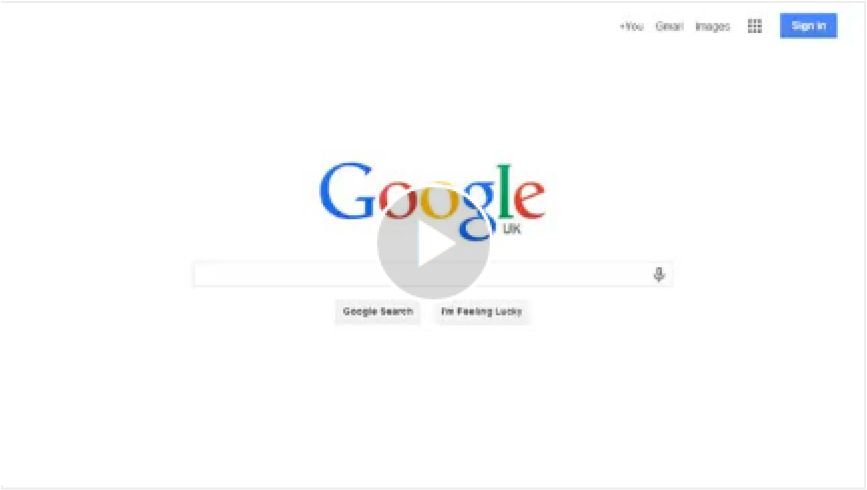

View transcript

Download video: standard

Using a scientific literature database

7 comments

Heather Worlledge-Andrews gives an overview of how to use PubMed, a very useful and freely available literature database in your research. You might find this useful in the following steps.

© University of Glasgow

Learning activity – short written assignment.

Learning type – production, learners are asked to articulate their understanding of the key concepts surrounding the links between epigenetics and cancer. They are provided with links to several open access resources on the topic and encouraged to search for more. Learners are encouraged to list the resources used in writing their assignment.

Assignment

You've now heard and read a bit about future directions in epigenetic research in cancer. However, I'm sure you will be aware that there are many unanswered questions. We've outlined a key question in epigenetic research in cancer below, and we would like you to carry out some research of your own.

Here is the question:

What do we know about how epigenetic regulation goes wrong in cancer and what types of targeted treatment could arise from our knowledge of epigenetic de-regulation in cancer?

We want you to write a 300 word assignment in answer to the question based on your own research. Your target audience should be a fellow learner on this course. At the end of it make a note of the resources which you used and include a link if these are online resources so that your reviewers can visit these websites and evaluate their reliability. This is an important part of your assignment to remember, and your reviewer will comment on your choice of resources in the feedback. Note that although 300 words seems quite short, we've purposely made it this length to help you to learn to write concisely - a key skill in science. **Your list of resources and associated web links are excluded from your final word limit.**

Research and resources

You've heard Heather talking about searching PubMed Central already. We've done a basic search on the question using this database and come up with a couple of interesting papers - you will find links to them below, at the end of the article.

Don't feel you have to read the whole paper from start to finish - use the subheadings to pick out the sections which are most useful to you.

Feel free to use these papers or, if you find them a bit too detailed, to do your own research using whichever resources you find most helpful. Some other potentially useful resources are listed at the end of this article.

If you do use an online resource consider its reliability, authority and its currency (how up-to-date) it is. Remember to include a web link to any online sources that you use at the end of your assignment so that your reviewer can view these resources. These links (and any other references) will be excluded from your word count.

Learning activity – peer review of other learners' assignments.

Learning type – production, learners are asked to review their peers' assignments using a rubric.

Review your colleague's work

Now you have investigated and written your own answer to the question, in this step you will review your colleague's work. This may be the first time you have done anything like this but don't worry, you have the guidelines below to help you. Try to give constructive feedback, highlighting areas which have been done well in addition to making suggestions about what could be improved. We hope you find it a useful exercise.

Occasionally the system may offer you for review something that may have been submitted in haste or error by a fellow learner. If this happens, provide polite brief feedback, submit it, and, request a new assignment by clicking here for a new assignment. You should then be able quickly to move on to review a different participant's work.

ASSIGNMENT GUIDELINES

You're going to be asked to give feedback on the following aspects of the author's assignment:

- What did you like about the author's work?
- Had they carried out research using reliable resources and had good use been made of these? Did they cite the sources?
- How might the author improve the communication of their key ideas?
